# Direct Fabrication of a Copper RTD over a Ceramic-Coated Stainless-Steel Tube by Combination of Magnetron Sputtering and Sol–Gel Techniques

**DOI:** 10.3390/s23125442

**Published:** 2023-06-08

**Authors:** Aitor Bikarregi, Santiago Dominguez, Marta Brizuela, Alejandra López, Ana Suarez-Vega, Cecilia Agustín-Sáenz, Micael Presa, Gabriel A. López

**Affiliations:** 1 Tubacex Innovación SL, 48160 Derio, Spain; alopez@tubacex.com; 2 Physics Department, Faculty of Science and Technology, University of the Basque Country, Barrio Sarriena s/n, 48940 Leioa, Spain; gabrielalejandro.lopez@ehu.eus; 3 Tecnalia Research & Innovation, 20009 San Sebastián, Spain; santiago.dominguez@tecnalia.com (S.D.); marta.brizuela@tecnalia.com (M.B.); ana.suarez@tecnalia.com (A.S.-V.); cecilia.agustin@tecnalia.com (C.A.-S.); 4 Tubacoat SL, 48160 Derio, Spain; mpresa@tubacex.com

**Keywords:** thin film, RTD, copper sensor, magnetron sputtering, sol–gel, tube

## Abstract

Reducing the economic and environmental impact of industrial process may be achieved by the smartisation of different components. In this work, tube smartisation is presented via direct fabrication of a copper (Cu)-based resistive temperature detector (RTD) on their outer surfaces. The testing was carried out between room temperature and 250 °C. For this purpose, copper depositions were studied using mid-frequency (MF) and high-power impulse magnetron sputtering (HiPIMS). Stainless steel tubes with an outside inert ceramic coating were used after giving them a shot blasting treatment. The Cu deposition was performed at around 425 °C to improve adhesion as well as the electrical properties of the sensor. To generate the pattern of the Cu RTD, a photolithography process was carried out. The RTD was then protected from external degradation by a silicon oxide film deposited over it by means of two different techniques: sol–gel dipping technique and reactive magnetron sputtering. For the electrical characterisation of the sensor, an ad hoc test bench was used, based on the internal heating and the external temperature measurement with a thermographic camera. The results confirm the linearity (R^2^ > 0.999) and repeatability in the electrical properties of the copper RTD (confidence interval < 0.0005).

## 1. Introduction

In an industry that is increasingly moving towards a digital form of business, the need to be connected to its production processes is increasing. In the Industry 4.0 era, the acquisition of the information regarding the status of these processes is highly valued. In sectors as diverse as chemical, power generation, or food, the critical parameters of both the process and the equipment must be monitored during a large part of the production process [1,2,3,4,5,6]. For a long time, the interest of companies in these and other sectors has focused on knowing the state of the fluids that circulate inside the pipes throughout their plants and equipment. Continuous monitoring prevents critical system failures, avoids leaks and energy losses, and thus helps keep costs down [7,8,9,10]. Meanwhile, the interest of the companies supplying this equipment has been to offer this service, justified by the interest it arouses.

However, most of the monitoring methods on the market are limited by their size or their high degree of invasion in critical processes. For this reason, in recent years, sensorisation and monitoring have evolved towards less invasive and more embedded methods [11,12,13]. Their benefits include maintaining the integrity of the pipes, thus ensuring greater safety, and ultimately lower installation and maintenance costs [14,15]. Among the existing methods (ultrasound, Eddy current, thermography, etc.) thin-layer sensors have the advantage of being able to be installed in areas which are difficult to access, in addition to presenting a faster response to changes in the system [16,17,18].

**Figure 1:**
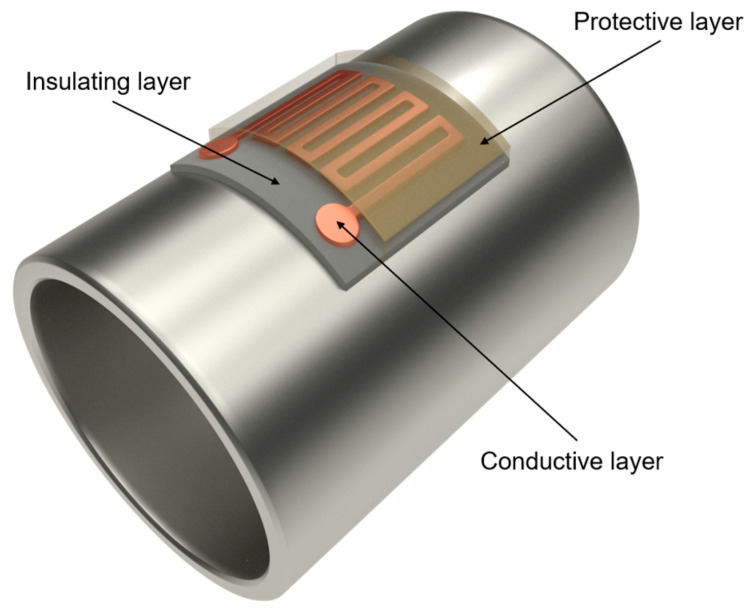
Layout of the temperature sensor.

As is known from the literature, a resistive temperature detector (RTD) is a device that changes its electrical resistance value according to the temperature, and it can be mainly classified in three categories: wire-wound, coiled elements, and thin-film. These sensors usually present better accuracy compared with other temperature sensors such as thermocouples. In the case of a sensor based on a thin-film RTD, this can be composed of various thin layers. A typical example of a multilayer sensor for a tube may have the following configuration (Figure 1):

- A layer of electrical insulator on the surface of the stainless-steel tube. Its function is to avoid electrical contact between the sensor layer and the tube.
- An electrically conductive or sensing layer, which is the RTD itself. This metallic layer has a varied electrical resistance as the temperature changes.
- A protective layer to protect the sensor layer from degradation phenomena such as high-temperature oxidation.

Therefore, in the context of this work, the RTD designation will refer to the sensor layer; that is, the copper layer. Meanwhile, the device made up of multiple layers will be defined as the temperature sensor.

Concerning the sensing layer, platinum is commonly used as a sensing material due to its excellent corrosion resistance and electrical response with good linearity for wide ranges of temperature [19,20,21,22,23], but its use implies a high economic impact in its industrial production. As a cheaper alternative, other materials have been studied, such as nickel [24,25,26,27] or aluminium [28,29]. In some of these studies, transfer processes of the pattern to a curved substrate have been carried out, requiring complex methods and several materials.

In the current contribution, a copper RTD for ceramic-coated stainless-steel tubes is introduced. In the past, few works reporting Cu-based [30,31] and combined Ti–Cu [32] and Cu–Ni [33] systems have been published, although the temperature range covered in those works is significantly lower. Details on the fabrication of a copper layer and a simple method patterning are provided, as well as the results of electrical tests carried out on a home-made prototype.

## 2. Background

### 2.1. Design of the Sensor Layer

An RTD needs an electrical circuit through which electrons circulate, usually made of a metal that will see its electrical resistance changed with the variation in temperature. By measuring this electrical resistance change, it is possible to measure the variation in temperature. In the case of metals, the electrical resistance increases with increasing temperature [34].

The model that currently best explains the behaviour of metals in solid structures is the band theory, being based on molecular orbitals and approximating the quantum state of a solid. Having a high number of valence orbitals, the energy levels together are considered to form continuous bands. Thus, two types of bands are differentiated: valence bands, where all the valence electrons of the atoms are found; and the conduction bands, where free electrons are found and responsible for conducting the electric current. In the case of a conductive material, these two bands will overlap or the gap between them will be minimal, allowing the electrons to jump between the bands. As the temperature of the metallic solid increases, the number of phonons generated within the material increases and they collide with the electrons, causing them to scatter. At the macroscopic level, this leads to an increase in the electrical resistivity of the material.

It is known that the resistance at room temperature (20–25 °C) of a circuit depends on its resistivity at that temperature (*ρ*), and the geometric parameters of the circuit (*l*: length, *A*: cross-section), for a uniform material and a constant cross-section:

(1) $$R_{0}=\rho \;\left(l/A\right)$$

For a wide temperature range, the following linear relationship between resistance and temperature holds, where *α* is the temperature coefficient of resistance:

(2) $$R_{T}=R_{0}\left(1+\alpha \left(T-T_{0}\right)\right)$$

Regarding the design and production of thin film sensing layers, generally, it is preferred to start with a high electrical resistance value at room temperature, so that it is possible to minimize the electrical noise in the measurement. From Equation (1), it is concluded that for the electrical resistance to increase, it is necessary to increase the length of the circuit and/or decrease its section. The latter happens if, in the case of a circuit with a rectangular section, both the channel width and the thickness are decreased. However, these three parameters (length, channel width, and thickness) are limited by total circuit size, photolithography, and material deposition technique, respectively. It is necessary to adjust these parameters to obtain the optimal design of the sensing circuit. Taking this into account, the design and layout of the RTD was made using computer software (EAGLE, from Autodesk).

### 2.2. Base Materials

As a worldwide manufacturer and distributor of seamless tubes of a wide variety of stainless steels and nickel-based alloys, Tubacex has a wide network of experts who are knowledgeable about industrial processes and their requirements, as well as the needs they demand [35,36]. Thus, after an exhaustive study by its highly qualified metallurgical staff, the candidate materials for a given application can be selected.

For this work, due to commercial interests, 310S steel was chosen, making use of both tube pieces and flat sheets. This material is used, for example, in Waste-to-Energy (WtE) or biomass boilers, because it has very good resistance to corrosion at high temperatures and is cheaper than other options (where the chromium and nickel content are what weigh more in cost). For the environments of the aforementioned applications, where many chlorinated compounds are generated and, therefore, corrosion in austenitic steels above 500 °C can be very fast, this stainless steel is covered with a special coating that will be discussed in the next section.

### 2.3. Isolation Layer

In order to avoid the electrical contact between the metallic pipes and the sensing layer, an electrical isolation of the surface of the pipe is needed. Some authors have used thin-film layers to isolate a metallic substrate via reactive magnetron sputtering with SiO_2_ and Al_2_O_3_ [37,38], or YSZ/Al_2_O_3_ multilayered films [39]. Others have used thermally sprayed alumina [40] and sol–gel deposition, or have applied Chemical Vapor Deposition (CVD) to fabricate SiO_x_ films [41]. In this work, an advanced thick ceramic coating was applied. This material is a commercial product from the company Tubacoat SL [42], which is part of the Tubacex Group. What is sought with Tubacoat is to minimize the adhesion of any material thanks to its inert composition (Table 1), thus lowering maintenance costs and times. In addition, the lifetime of the tubes is extended by not exposing the metal to fouling or corrosive atmospheres and high temperatures.

## 3. Experimental Section

### 3.1. Isolation Layer Preparation

Due to the anti-adherent property (low roughness and chemically inert borosilicate composition) that the ceramic coating presents on its surface, the adhesion of any type of material on it is highly compromised regardless of the thickness applied and the technique used. Therefore, to improve this property to acceptable values, it was necessary to increase the effective surface roughness by a process of shot blasting. This modification was carried out by means of particles of white corundum (particle sizes F220 and F500) and different surface finishes were obtained over the ceramic coating. In this process, 3 bar of pressure shot was used while a shot blasting distance from the nozzle to the substrate surface of 0.5 m was kept.

### 3.2. Sensor Layer

The sensor layer was made of copper due to its linearity in the electrical response [30,31] and the low cost, compared to other materials such platinum or gold. In the case of copper, the temperature for which its linearity is maintained according to Equation (2) ranges from −200 °C to 260 °C [44]. In this work, its cryogenic properties will not be tested, not even below room temperature. Therefore, the operating range was reduced from 25 °C to 250 °C. For the terminal connections of the RTD, 1 mm diameter cables of nickel-plated copper and a high-temperature epoxy paste were used.

Before fabricating the sensing layer, its theoretical resistance value at room temperature was calculated (Table 2) according to Equations (1) and (2) after defining the geometrical parameters expected from its fabrication.

The coatings corresponding to the sensor layer were deposited via PVD magnetron sputtering in two different Cemecon units: CC800/8Plus and CC800HiPIMS (batch configuration). The first one uses four mid-frequency (MF) power supplies (Advance Energy PE II). In the case of the unit CC800HiPIMS, it presents four high-power impulse magnetron sputtering (HiPIMS) cathodes and two additional cathodes in direct current (DC) mode. The targets used were Cu (99.9%) in two different rectangular sizes: 200 × 88 mm and 500 × 88 mm (height × width) regarding the unit used (CC800/8Plus and CC800HiPIMS, respectively).

To study the influence of the roughness of the ceramic substrate on the adhesion properties of copper over it, a copper layer was deposited with the MF power supply on different surface finishes. Subsequently, another study was carried out, but in this case to determine and compare the conditions for the synthesis of copper. The experimental conditions for the adhesion and sensor layer studies are gathered in Table 3.

Before the deposition of the copper coating or layer, the samples were cleaned with acetone, and then immersed in ethanol and cleaned with an ultrasound bath. Finally, the tubes were dried with compressed air. Pieces of silicon wafer (100) were also cleaned and introduced into the vacuum chamber to measure the thickness and the X-ray diffractograms.

Using these diffractograms and the Scherrer equation, the mean size of the ordered crystalline domains for all the copper coatings over silicon samples were calculated according to the following equation:

(3) $$D=\frac{K\;\lambda }{\beta \;\cos\theta }$$

where *K* is the shape factor (=0.9), *λ* is the X-ray wavelength (=0.15406), *β* is the line broadening or full width at half maximum, and *θ* is the Bragg angle. The width and form of the peaks of a diffractogram depend on instrumental factors and the sample microstructure. Therefore, under the same instrumental conditions, the microstructure of different samples can be compared.

In both sputtering equipment, the samples were mounted on a planetary carrousel and introduced into the vacuum chamber with a rotary table. After achieving a minimum base pressure of 6 mPa, a process of heating was carried out at 425 °C over 2 h (4 kW) to bake the vacuum chamber, the substrate holders, and all the inner components. This was carried out by applying 4 kW to the resistance elements installed in the inside of the chamber. The power applied to the cathodes was 0.5 kW during 1 h of the deposition process. During this time and due to the batch configuration (double rotation), the surfaces of the samples were exposed alternatively to the plasma sources. The working gas used was Argon 280 sccm with a deposition pressure of 625 mPa.

To study the influence of temperature on the structure of copper, certain samples underwent heat treatment, also known as annealing. This process was carried out in the same vacuum chamber as the PVD equipment, in vacuum conditions (lower than 10 mPa) for 4 h at about 450 °C.

**Figure 2:**
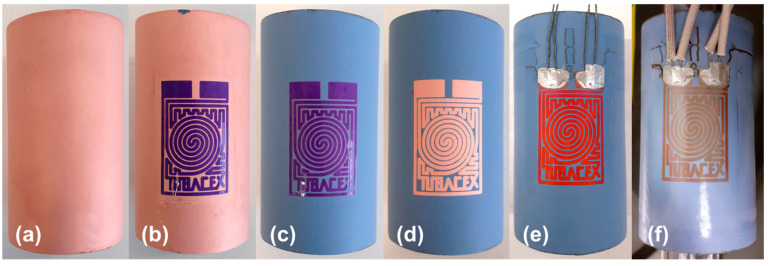
Fabrication of the sensor. (**a**) Copper deposition. (**b**) Mask obtention along the tube. (**c**) Etching with ferric chloride. (**d**) Elimination of the mask. (**e**) Welding of the cables. (**f**) Protection with sol–gel. Masks designed with Eagle (Autodesk, Inc., San Rafael, CA, USA).

After the deposition of the sensor layer, it is necessary to generate an electrical pattern on it. Therefore, the layer will act as an RTD (Figure 2).

In a preliminary step, a negative version of the electrical pattern was printed on a transparent film to act as a light mask in a modified photolithographic process. First, a negative photoresist paper piece was put over the surface of the copper deposited on the pipe (over the isolating layer) and was heated to improve the adherence to the surface. After this, the system was masked with the developed negative pattern mask and UV radiation was applied. By doing so, the parts of the photoresist paper that were exposed to light became insoluble, while the masked parts were soluble for a developer with a specific concentration of a solvent. To eliminate those uncured parts, the piece of tube was then immersed in a low concentrated sodium carbonate dissolution (1.25% in Vol.). Once a good-quality mask with the desired pattern was obtained in the surface (Figure 2b), the rest of the copper material was taken away (Figure 2c). This was achieved by etching it with an aqueous ferric chloride dissolution (500 g per 1 L of water). Finally, the cured mask was eliminated (Figure 2d) by a highly concentrated sodium carbonate dissolution (>5% in Vol.).

To stablish electrical connection with the sensing layer, four wire configurations were made to obtain an accurate measurement of the electrical resistance. Considering the temperature range (20–250 °C) that was defined in the beginning of the development, a high-temperature bi-component epoxy solder was used (G6E-HTC^TM^ High Temperature Carbon-Filled Electrically Conductive Epoxy, G6-Epoxy^TM^), observing a colour change in the copper layer after the curing of the epoxy (Figure 2e).

### 3.3. Protective Layer

To protect the copper RTD from deterioration due to handling with bare hands and to prevent oxidation at high temperatures (in this work, more than 200 °C), it was necessary to apply a protective coating to the system formed by the isolating and sensor layers. From a commercial point of view, it was a desirable option to display the logo on the RTD, so transparent silicon oxide (SiO_2_) was deposited on the system via two techniques. Table 4 summarizes the principal parameters of both techniques, along with the obtained mean thickness.

One of them was PVD magnetron sputtering, but with its reactive variant; that is, introducing a gas (oxygen, around 50 sccm) that reacts, in this case, with silicon. A Cemecon CC800/8Plus unit was used with closed-loop mode, voltage monitoring, and SpeedFlo processing via Gencoa using MF power supplies (Advance Energy PE II). For the deposition process, 2 kW was applied to the heating elements and the cathodes were set up with 3.5 kW. A poisoning set point of 40% was established.

The other chosen technique was sol–gel, in which a formulation based on a molar ratio of 1:2:5 of TEOS, MTES, and nitric acid, respectively, in ethanol (as the solvent) was deposited via dip-coating. First, the solution was prepared with the mentioned reagents, which has required a process of hydrolysis and condensation to form the sol. Subsequently, the sample was introduced into the sol, and after a controlled speed extraction (using an automatic robot), the solvent of the xerogel layer formed on the surface of the sample was evaporated at room temperature. A curing time of one hour was necessary and different temperatures were applied and compared (200 °C, 300 °C, and 450 °C). The selection of this technique configuration was made considering that in this way the coverage is maximize since it is an immersion technique.

### 3.4. Film Characterization

Properties such as roughness or film thickness were measured using contact profilometry using a Dektak 150 (from Dektak company) profilometer. For a rapid evaluation of the metallographic preparation of the sensor, a Leica DM6000M optical microscope was required. A field emission scanning electron microscope (FESEM) from the ZEISS Sigma family was used to measure and compare the thickness of the sensing layer between silicon wafer and pipe deposition. In the case of the semi-quantitative chemical composition measurements, an energy-dispersive X-ray spectroscopy (EDS) technique was used, with an X-ray detector from Bruker attached to the FESEM equipment (working distance of 9 mm and a voltage of 15 kV). X-ray Diffraction (XRD) (Bruker D8 Advanced) was utilized to measure the crystalline phases of the coatings by using Cu-Kα radiation (*λ* = 1.544 Å) at a glancing angle of incidence of 7.5° (step time = 1 s, step = 0.03°).

### 3.5. Electrical Characterization

**Figure 3:**
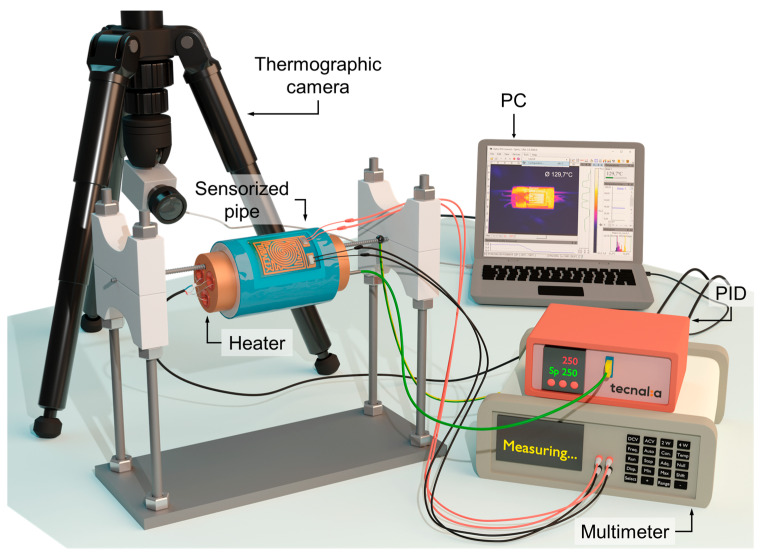
Custom test bench.

To characterize the temperature sensors on the tube in an adequate way, a custom test bench was developed (Figure 3). The heating system uses three resistive cartridge elements inserted into a cylindrical copper holder (with the same diameter as the internal one of the tubes tested) that heat the tube from the inside. The electrical power applied to these resistors is controlled by a commercial PID controller (proportional–integral–derivative controller) and a thermocouple placed next to the three resistors.

On the other hand, the measurement of the temperature of the surface of the pipe was carried out using a thermographic camera (Optris PI) and the known emissivity of Tubacoat [43]. In this way it was achieved a more accurate measurement on the surface. The electrical resistance is acquired with a multimeter (Keysight 34461A) with an acquisition rate of 1.9 s/point. These two measurements, the external temperature of the tube and the electrical resistance, are recorded and saved using a custom LabVIEW program installed on a computer.

## 4. Results/Discussion

### 4.1. Adhesion Study

Due to the low roughness of the Tubacoat isolating layer (*R*a < 0.04 µm), the first attempts to deposit copper showed a total lack of adhesion. In consequence, it was necessary to perform an optimization process of this property by means of surface modification. Thanks to the high thickness of the isolating layer, the best option was to increase the surface roughness by means of shot-blasting processes using two types of particle size: F500, with an average particle diameter of 12.8 µm (FEPA Grain Size) (*R*a after blasting 0.19–0.22 µm); and F220, with a mean diameter of 58 µm (*R*a after blasting 1.1–1.3 µm), as is shown in Table 5.

**Figure 4:**
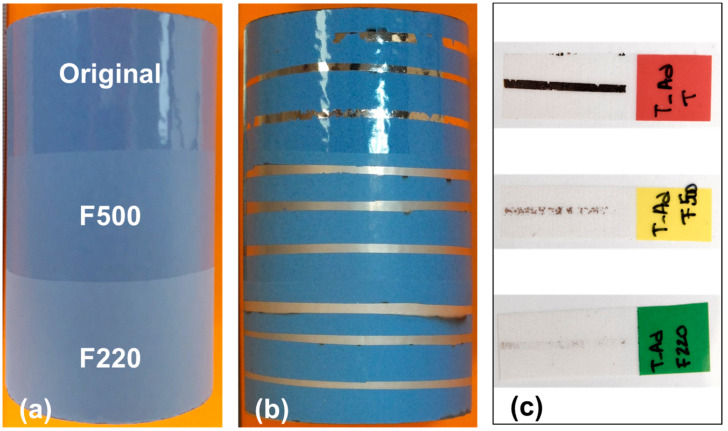
Adhesion study over Tubacoat. (**a**) Before copper deposition. (**b**) After copper deposition. (**c**) Scotch tape test.

In addition, the synthesis conditions applied in the magnetron sputtering processes was optimized, heating the substrates (with a minimum of 500 W applied) to increase the energy and the surface diffusion of the copper particles that arrive at the substrates. As can be seen in Figure 4, three copper lines were deposited on the three different surface finishes to carry out the test. To evaluate the adhesion of the copper, a qualitative method was performed using an adhesive tape on each copper line, the F220 particle size treatment being the best option with minimal material transferred from the coating to the adhesion tape. Therefore, in consequence, all the sensor layers developed in this work were produced using this shot-blasting treatment for the isolating layer.

### 4.2. Sensor Layer Study

Temperature is one of the most important synthesis parameters, not only for properties such as adhesion, but also for microstructure, mechanical, or electrical properties. In this work, the effect of synthesis temperature on the electrical properties, derived from the type of structure they present, was studied.

On one hand, the HiPIMS technique, in general terms, is based on developing short duration pulses on the targets, maintaining the equivalent RMS power. The main consequence is the generation of a higher density of ionized species from the target, so, it is possible to achieve much denser layers, and in terms of mechanical properties best performance compared with classical DC mode [46,47]. After depositing copper via HiPIMS on a silicon wafer at a low temperature (HiPIMS Low T) and carrying out the appropriate characterization, this sample underwent a vacuum heat treatment for 4 h at about 450 °C (HiPIMS Low T + Anneal).

On the other hand, MF technique presents great versatility, and it can sputter materials with low electrical conductivity or deposit metal oxides or nitrides in a reactive synthesis process. In this case, the sample was also deposited at low temperature (MF Low T) and then thermally treated under the same conditions (MF Low T + Anneal). However, in addition, a third sample was manufactured by providing energy through the heating resistances located inside the vacuum chamber during growth or synthesis of the copper layer, so the effect of temperature could be studied (MF High T).

**Figure 5:**
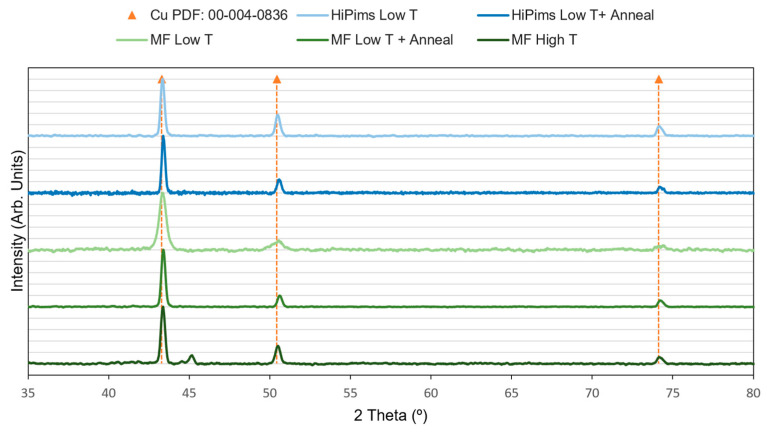
The XRD image of Cu thin films.

**Figure 6:**
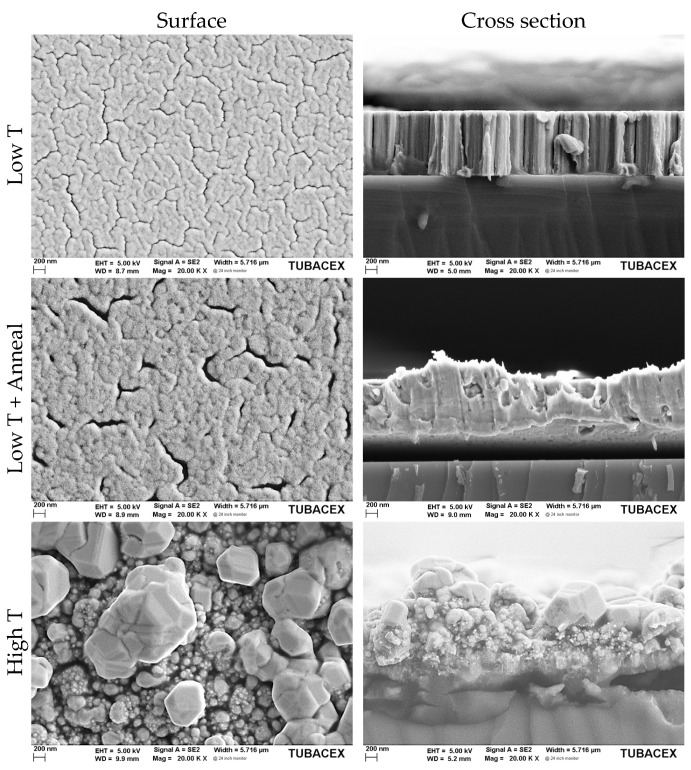
Copper thin films deposited at different conditions in MF.

Figure 5 shows the diffractograms of both the samples deposited via HiPIMS and via MF. Besides observing a sharpening of the peaks in those samples with heat treatment, two smaller peaks around 45° are observed in the high-temperature MF sample. According to the literature, these peaks correspond to the Cu_3_Si intermetallic phase, which is formed due to the interdiffusion of these elements at high temperatures [48,49,50]. This observation is in good agreement with the FESEM images of this sample in Figure 6, where an interaction between copper and silicon can be seen in the cross-section image.

Table 6 lists the mean size of the ordered crystalline domains (*D*) calculated by full width (*β*) at half maximum (2*θ*). It can be observed clearly that increasing temperature leads to growth of the crystals, in good agreement with data reported previously [51]. Even so, a smaller increase due to the annealing process is also deduced for the case of HiPIMS, because this technique applies enough energy to the copper to make it grow (small pulses, but high energy) even at low temperature [52,53]. On the other hand, it is evident that the coating obtained by the MF unit needs temperature to increase its grain size, either by applying it during the synthesis or in a subsequent treatment. Finally, for these materials (Cu and Si), there is a temperature limit to be applied, since diffusion effects occur which may not be of interest to maintain the good performance of the RTD.

For better understanding of the high increment on the domain size of the MF samples, a microstructural analysis was carried out using an FESEM microscope. Figure 6 shows the top view of the sample surfaces and their cross-sections. An evident coarsening of the grains can easily be observed considering the larger voids in the Low T + Anneal sample but in lower density than in the Low T sample. A possible correlation between this fact and the grain growth promoted by applying a tempering process is attributed to this observation. In addition, the large grain size induced by temperature during growth is observed for the High T sample, even though it has the worst appearance in terms of continuity.

In these results, the positive effect of temperature during synthesis was observed since it helped to obtain stable layers. In the electrical response of the sensors shown hereafter, the technique with which the copper layers were deposited will not be a variable of interest, and therefore it will not be referred to. Some were made with HiPIMS, and others with MF with heating during or after the process. That is, in all samples characterized electrically, only stable copper layers with a similar crystal size were investigated.

### 4.3. Electrical Response

Over a piece of pipe coated with Tubacoat and shot-blasted on the surface with F220, the sensor layer of copper was manufactured satisfactorily. Subsequently, photolithography was carried out on the copper with the designed mask and four cables were soldered with the epoxy paste. After curing the paste, it was covered with the coating TTW001 (sol–gel technique, Table 4) and cured at 200 °C for 1 h.

**Figure 7:**
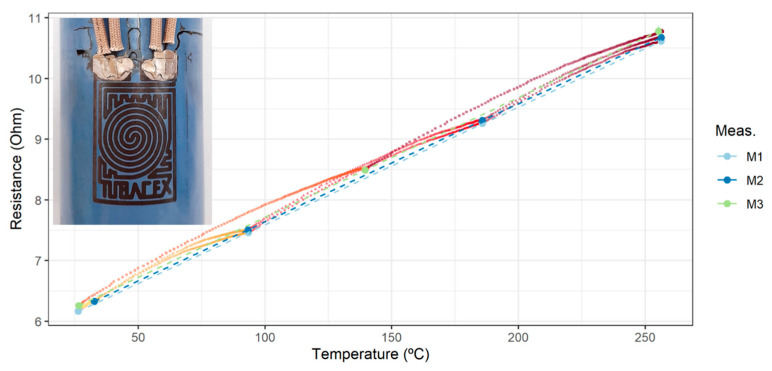
Electrical characterization for the copper RTD with TTW001 protection. Error bars *100.

Figure 7 shows the results of the electrical characterization. For each of the measurements (curves M1 to M3), several points were taken in steady state throughout the heating process in the temperature range between room temperature and 250 °C. The transitory values of this heating process are indicated by the colour gradient that goes from yellow to maroon. Meanwhile, each stationary point corresponds to the mean value of 10 min of acquired data. The confidence interval for these calculations in the case of the electrical resistance is also plotted. Since these errors are so small, they cannot be seen on the graph even after increasing them 100 times. This is indicative that the steady state was reached. Additionally, linear regressions were plotted for each of the measurements (R^2^ > 0.999 for the three of them). From the plots of the complete heating test, a fast response of the RTD to changes in temperature is deduced.

To test the thermal stability of the sensor in the limit of the temperature range, it was maintained for at least 1 h at around 250 °C. The results show a very small increase of the resistance value at room temperature (*R* [20 °C]) after the whole set of measurements, with the corresponding annealing-like treatment, in comparison with the starting resistance at room temperature. A slight increase in the slope after each test can also be observed. This is a very good feature of the sensor, because it is very stable even after a few cycles. Later in the text, these results together with the value of the temperature coefficient (TCR) are graphed. It is worth mentioning that a slight colour change of the sensor was observed after the thermal tests.

**Figure 8:**
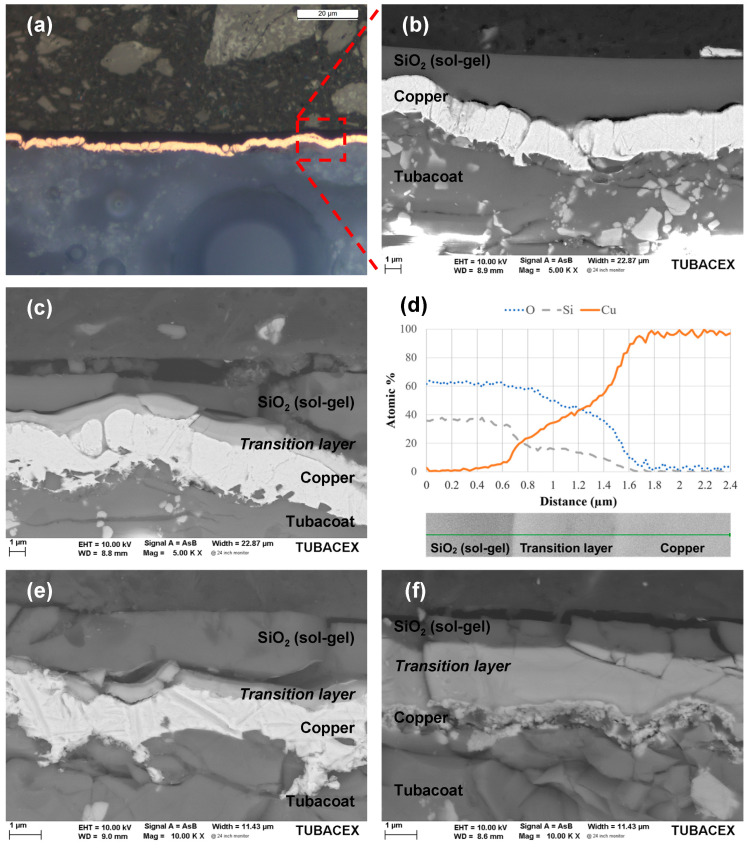
Cross-sections of different curing temperatures of the sol–gel applied over the sensing layer. (**a**,**b**) Optical microscope and SEM images, respectively, of the sensor after a 200 °C curing. (**c**) Same sensor after all the electrical characterization. (**d**) Compositional analysis of the diffusion layer. (**e**) SEM image of the sensor after a 300 °C curing. (**f**) SEM image of the sensor after a 450 °C curing.

To see the changes that occurred during the electrical characterization, the sensor was cut crosswise, and, after an exhaustive and careful metallographic preparation, it was characterized by optical microscopy and FESEM. Figure 8 compares the microstructure of the sensors before and after an electrical test. Figure 8a,b refer to a RTD that has just passed a curing of 200 °C for the TTW001 protective coating. In the enlargement shown in Figure 8b, it is observed that the thickness of the copper is about 2.5 µm throughout the whole sensor layer. As the Magnetron Sputtering is a conformal synthesis method, the thickness is very uniform along the surface of the tube and replicates the shot-blasted ceramic surface. Meanwhile, in the case of the sol–gel layer, as it is not a conformal fabrication method, the measured thickness ranges between 1 and 5 µm, covering all the copper RTD and smoothing out the roughness in the surface.

Figure 8c shows an interphase region between the sensor layer and the protective coating that appeared after the electrical characterization of Figure 7. A semi-quantitative EDS line profile acquired on this interphase region is depicted in Figure 8d. It clearly shows the appearance of an interdiffusion layer occurring upon the thermal treatment experienced during the curing/electrical characterization. Therefore, the evidence indicates that between 200 °C of curing and 250 °C of maximum working temperature, there is a threshold temperature for which the copper and TTW001 layers interact.

To check the influence of the curing temperature of the TTW001 coating, this value was increased above the defined working temperature of the sensor, choosing curing temperatures of 300 °C and 450 °C. However, after performing the FESEM characterization, not only was the formation of this diffusion layer confirmed in both cases (Figure 8e,f), but also a significant thickness increase with increasing temperature. Furthermore, in the case of 450 °C, the copper layer was almost completely consumed. Therefore, the sol–gel formulation (TTW001) proposed in this work is limited to be used in applications with service temperatures below 200 °C to avoid the degradation of the RTD.

Looking for an alternative to the sol–gel method, a protective layer of silicon oxide was deposited using the PVD-RMS technique with a thickness of 5 µm on a new RTD. The coating referenced by TTD001 (Table 4) was applied at a temperature lower than 300 °C. Figure 8 shows the curves M0′ and M1′ that correspond to the electrical response of the first two measurements that were made on this sensor. In addition, this graph includes the thermal stability tests of the sensor at a temperature of around 250 °C for at least 1 h. The linearity of the resistance of the copper RTD as a function of the temperature (R^2^ > 0.997) and its fast response time during transient periods is again observed. However, it is observed that the electrical resistance at high temperature is less stable during the first heating than during the second one.

**Figure 9:**
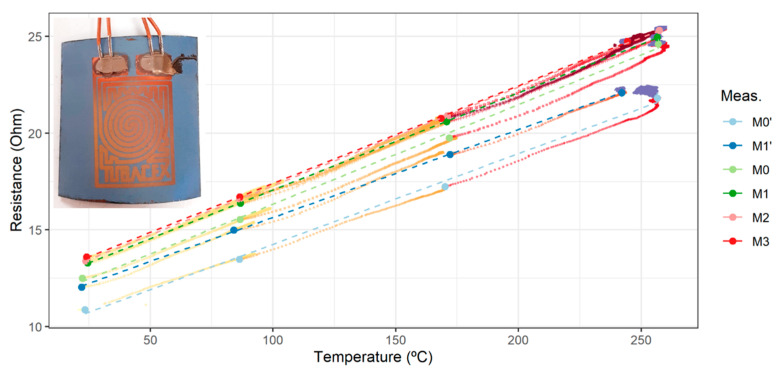
Electrical characterization for the copper RTD with TTD001 protection by PVD-RMS. M0′ and M1′ refer to the characterization after the first protecting coating. M0 to M3 correspond to the response after the second coating. Error bars *100.

After observing an increase in resistance at room temperature after the first test (from M0′ to M1′), it was decided to proceed with the deposition of a second layer of silicon oxide in order to seek an improvement in its electrical stability. The results of the electrical characterization are also shown in Figure 9 (curves M0 to M3). In this case, the same phenomenon observed in M0′ is also observed for the M0 test, again detecting an unstable behaviour at high temperature, but a significantly greater stability and repeatability were detected in the subsequent measurements M1, M2, and M3 (R^2^ > 0.999).

**Figure 10:**
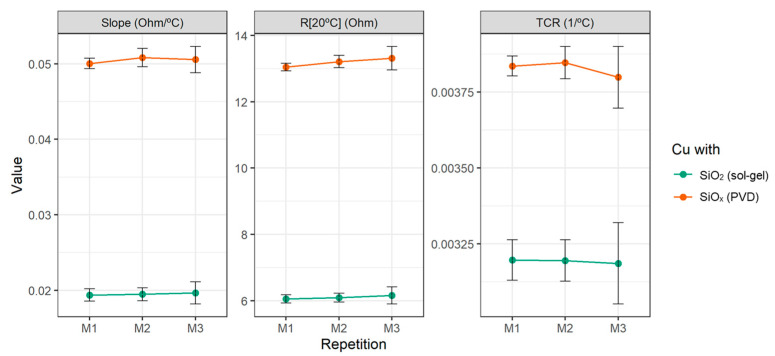
Slope, R [20 °C], and TCR of the copper RTD after three measurements. Error bars *100.

The electrical parameters of interest for these curves are found in Figure 10 where they are compared with those of the sensor produced with the sol–gel method. As in the previous results, the confidence interval was multiplied by 100 for better interpretation. It can be seen how the three characteristic curves (M1, M2, and M3) of the sensor were obtained for each protective material, with the same slope, R [20 °C], and TCR, all indicating the good repeatability of the copper RTD in the working range. The confidence interval was calculated for the mean values of the slope and TCR, in both sol–gel and PVD cases, resulting in values below 0.0005 and 3 × 10^−5^, respectively.

**Figure 11:**
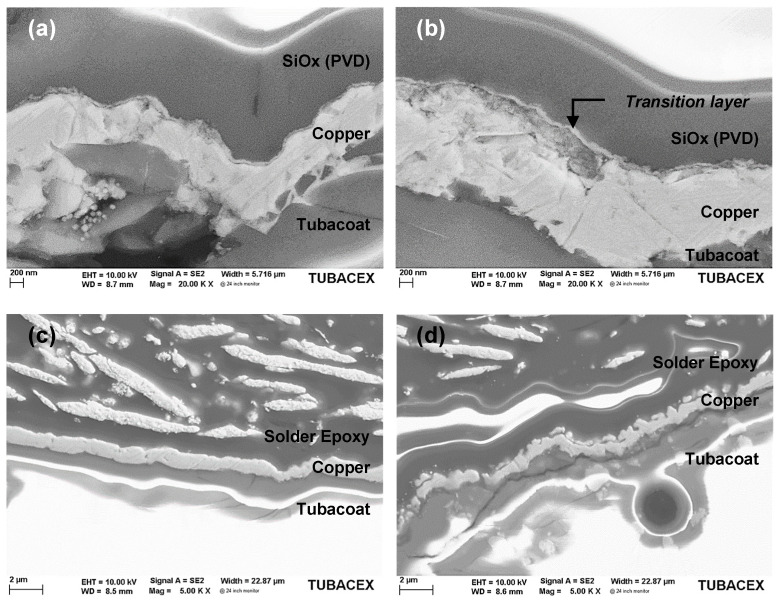
SEM images in transversal section of a copper RTD with TTD001 protecting layer. (**a**) and (**b**) Sensor zone. (**c**) Thin solder epoxy zone. (**d**) Thick solder epoxy zone.

To better understand the phenomenon behind the increase in electrical resistance that occurs at high temperature, it was metallographically prepared in two cross-sectional areas of the tube sensor: one in the sensor itself and another in the part of the terminals. Images (a) and (b) in Figure 11 show the copper sensing layer on Tubacoat with the silicon oxide deposited on it. It can be seen how there is a very thin layer of copper separated from the rest of the sensing layer which belongs to the protective layer. It appears that there was diffusion from the copper through the protecting layer. EDS analysis of the protective layer SiOx shows a silicon/oxygen ratio of 30/70, with x equal to 2.33.

For the area of the terminals, the interaction of the epoxy with the copper was analysed (images (c) and (d)). A considerable difference in this interaction was identified depending on the thickness of the epoxy paste, observing how in image (c) that there is no apparent change in the copper layer (40 µm of epoxy); while image (d), which corresponds to the area with more epoxy paste (1 mm of epoxy), shows how the surface of the copper in contact with said paste was modified, probably during its curing.

The diffusion phenomena observed in this work between copper and the two protective layer options (silicon oxide both by sol–gel and by PVD) could be assigned to the high diffusivity of copper in silicon and silicon oxide. This problem becomes serious at temperatures above 200 °C and was reported previously, mainly in electronic applications [54,55,56,57]. The most common solution is to create diffusion barriers based on other materials between the implied layers (copper- and silicon-based), to prevent interaction between these two materials. Likewise, the temperature range in which this barrier is stable will be conditioned by the materials used to create it. Results were reported with the use of alloys with copper and other metals such as aluminium, calcium, or tantalum [58,59,60,61,62,63], or with various types of oxides, carbides, or nitrides [49,64]. To work at very high temperatures, high-entropy alloys were also been deposited at the interface of copper with silicon [65].

## 5. Conclusions

In this work, thin-layer copper resistive temperature detectors were fabricated over real pipes with a ceramic coating using the magnetron sputtering technique, observing the importance of temperature in the case of the MF power supply, and to a lesser extent for the HiPIMS. Therefore, considering the energy efficiency and the optimization of the number of steps, the HiPIMS technology seems more promising. After protecting the copper RTD with silicon oxide using sol–gel and reactive magnetron sputtering techniques and electrically characterizing it on a custom-made bench, the linearity of copper (R^2^ > 0.999) in the operating range between room temperature and 250 °C was confirmed. In addition, after several heating tests, the repeatability of the sensors was demonstrated by observing that the mean values of the slope and TCR have a confidence interval below 0.0005 and 3 × 10^−5^, respectively. Even so, the presence of a threshold temperature between the chosen materials (copper and silicon/silicon oxide) is evident, which would be around 190–200 °C. This requires optimization and incorporation of new materials between these two layers to extend the proper operating range beyond 200 °C.

## Figures and Tables

**Table 1:** Properties of Tubacoat [43].

General Composition of the Coating	Borosilicate Glass
Thickness range (µm)	100–150
Average roughness, *R*a (µm)	<0.04
Average of the maximum peak-to-valley height, *R*z (µm)	0.2
Emissivity, *ε*, at 20 °C [43]	0.89
Emissivity, *ε*, at 550 °C [43]	0.84

**Table 2:** Parameters for theoretical calculation of the resistance and its value.

Sensor Layer Geometrical Parameters	
Length (mm)	500
Channel width (mm)	1
Thickness (µm)	2
**Copper electrical properties at 20 °C** [45]	
Resistivity (Ω*m)	1.71 × 10^−8^
Temperature coefficient of resistance (1/°C)	0.00393
**Theoretical resistance (Ω)**	**4.278**

**Table 3:** Deposition conditions for the adhesion study and the sensor layer study.

Layer	Power Supply	Heating Output	Applied Power (W)	Thickness (µm)
Adhesion study	MF	0	500	1.2
Sensor layer study	HiPIMS	0	500–1000	1.9
	MF	0	500	1.1
		4000 W	500	1.4

**Table 4:** Principal parameters of the two transparent protective coatings.

Coating Name	Mean Thickness (µm)	Deposition Technique	Synthesis T (°C)	Curing T (°C)	Extraction v (mm/s)
TTW001	1–5	sol–gel	RT	200, 300, 450	575
TTD001	5.5	PVD-RMS	<300	-	-

**Table 5:** Roughness of different surface finishes.

Surface Treatment	Particle Diameter (FEPA)	Ra (µm)	Rz (µm)	Adhesion
Original	-	<0.04	0.2	Null
F500	12.8 µm	0.19–0.22	1.5–2.5	Very good
F220	58 µm	1.1–1.3	6.7–11.7	Excellent

**Table 6:** The mean size of the ordered crystalline domains.

Sample	β (rad)	2θ (°)	D (nm)	Increment *
HiPims Low T	0.0052	43.35	29	-
HiPims Low T + Anneal	0.0044	43.38	34	18%
MF Low T	0.0088	43.32	17	-
MF Low T + Anneal	0.0052	43.38	29	69%
MF High T	0.0055	43.38	27	59%

* Compared to Low T.

## Data Availability

The data that support the findings of this study are available from the corresponding author upon reasonable request.
